# Suppurative thyroiditis

**DOI:** 10.1002/ccr3.1463

**Published:** 2018-03-05

**Authors:** Mandeep Singla, Saurabh Gaba, Kanwar Bhinder

**Affiliations:** ^1^ Department of General Medicine Government Medical College and Hospital Chandigarh India

**Keywords:** Fluid collection, suppurative thyroiditis, thyroid gland, thyroid profile

## Abstract

Acute bacterial suppurative thyroiditis is an infrequent but life‐threatening medical emergency. It needs to be considered in a patient with painful thyroid enlargement. Therapy consists of appropriate antibiotics and drainage of abscess. The disease may prove fatal if diagnosis and treatment are delayed.

## Case Description

**Figure 1:**
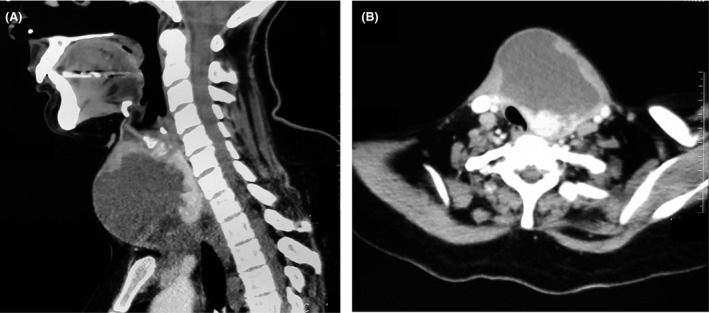
(A) Thyroid gland abscess extending superiorly to the submandibular region and inferiorly to the clavicular head. (B) Thyroid gland abscess causing mild compression of the trachea

A 40‐year‐old woman presented with a 1‐week history of high‐grade fever, odynophagia, and swelling in the right anterolateral part of the neck. On examination, her right anterolateral neck was swollen, warm, and tender. Hemogram revealed total leukocyte count of 13,200/mm^3^, with neutrophil count of 11,220/mm^3^. Computed tomography scan of the neck revealed 5 cm × 5 cm × 6 cm fluid collection in the right lobe of thyroid gland extending superiorly to the submandibular region, inferiorly to the clavicular head, medially involving the isthmus, and a part of the left lobe with mild compression of the trachea (Fig. 1A and B). Purulent fluid was aspirated from the collection, which grew methicillin‐sensitive staphylococcus aureus. Patient improved completely after ultrasound‐guided percutaneous drainage of the abscess and cefuroxime therapy. Thyroid profile showed FT3‐3.8 pg/mL (2.3–4.2), FT4‐1.4 ng/mL (0.89–1.76), and TSH‐0.65 *μ*IU/mL (0.35–5.5). Thyroid gland is resistant to infection because of its encapsulation, high iodide content, rich blood supply, and extensive lymphatic drainage 1. Suppurative thyroiditis usually develops in patients with pre‐existing thyroid disease (multinodular goiter or thyroid cancer), congenital anomalies like pyriform sinus fistula, and immunocompromised state 2. Therapy consists of appropriate antibiotics, drainage of abscess, and partial or total thyroidectomy.

## Authorship

All the authors made substantial contribution to the preparation of this manuscript and approved the final version for submission. MS: revised the manuscript for critically important intellectual content. SG: drafted the initial version of the manuscript and KB: acquired the images.

## Informed Consent

Informed consent has been obtained for the publication of this clinical image.

## Conflict of Interest

The authors have declared that no conflict of interest exists.
